# Urinary Biomarkers and Their Potential for the Non-Invasive Detection of Endometrial Cancer

**DOI:** 10.3389/fonc.2020.559016

**Published:** 2020-11-03

**Authors:** Kelechi Njoku, Davide Chiasserini, Eleanor R. Jones, Chloe E. Barr, Helena O’Flynn, Anthony D. Whetton, Emma J. Crosbie

**Affiliations:** ^1^ Division of Cancer Sciences, Faculty of Biology, Medicine and Health, School of Medical Sciences, University of Manchester, St. Mary’s Hospital, Manchester, United Kingdom; ^2^ Stoller Biomarker Discovery Centre, Faculty of Biology, Medicine and Health, Institute of Cancer Sciences, University of Manchester, Manchester, United Kingdom; ^3^ Section of Physiology and Biochemistry, Department of Experimental Medicine, University of Perugia, Perugia, Italy; ^4^ Department of Obstetrics and Gynaecology, Manchester University NHS Foundation Trust, Manchester Academic Health Science Centre, Manchester, United Kingdom

**Keywords:** urine, early detection, diagnostic biomarkers, endometrial cancer (EC), non-invasive (urine)

## Abstract

Endometrial cancer is the most common malignancy of the female genital tract and its incidence is rising in parallel with the mounting prevalence of obesity. Early diagnosis has great potential to improve outcomes as treatment can be curative, especially for early stage disease. Current tests and procedures for diagnosis are limited by insufficient accuracy in some and unacceptable levels of invasiveness and discomfort in others. There has, therefore, been a growing interest in the search for sensitive and specific biomarkers for endometrial cancer detection based on non-invasive sampling methodologies. Urine, the prototype non-invasive sample, is attractive for biomarker discovery as it is easily accessible and can be collected repeatedly and in quantity. Identification of urinary biomarkers for endometrial cancer detection relies on the excretion of systemic biomarkers by the kidneys or urinary contamination by biomarkers shed from the uterus. In this review, we present the current standing of the search for endometrial cancer urinary biomarkers based on cytology, genomic, transcriptomic, proteomic, and metabolomic platforms. We summarize the biomarker candidates and highlight the challenges inherent in urinary biomarker discovery. We review the various technologies with promise for biomarker detection and assess these novel approaches for endometrial cancer biomarker research.

## Introduction

Endometrial cancer (EC) is the most frequently diagnosed malignancy of the female genital tract and the sixth most common cancer in women globally (1, 2). The GLOBOCAN series of the International Agency for Research on Cancer reports a worldwide age-standardized incidence rate (ASR) of 8.4 per 100,000 women and mortality rate of 1.8 per 100,000 women, based on 2018 estimates from 185 countries (1). ASRs vary widely both across and within countries, from one to 30 cases per 100,000 women (2, 3). The highest incidence rates are reported in Western countries, particularly those with a high Human Development Index (HDI), where over 60% of all cases occur. Incidence rates are lowest in Sub-Saharan Africa, South Central Asia, and the Middle-East (2). In the United Kingdom, EC is the fourth most common female cancer with over 9,000 incident cases each year, and has in the past decade increased in incidence by almost 20% (4).

EC is commonly classified into two histological types based on a model that incorporates clinical, metabolic and epidemiological features (Bohkman’s dichotomous model) (5). Type I tumors are commonly low grade, estrogen driven tumors that are associated with obesity and a favorable prognosis. Type II tumors, by contrast, are high grade, estrogen independent tumors that are clinically aggressive and less strongly associated with obesity (5, 6). A molecular classification of EC by the Cancer Genome Atlas Research Network categorizes EC into four prognostically distinct subtypes: polymerase-epsilon (*POLE)* ultramutated, microsatellite instable, copy number low, and copy number high, and has been validated in multiple studies (7, 8).

Obesity is the strongest risk factor for EC and is estimated to be responsible for up to 40% of all EC cases (9, 10). Other EC risk factors include age, diabetes, hypertension, polycystic ovary syndrome, nulliparity, use of estrogen-only hormone replacement therapy, and tamoxifen (10, 11). Women may also have a familial predisposition to EC, in particular, those who carry a pathogenic variant in one of the DNA mismatch repair genes (Lynch syndrome) or the tumor suppressor gene-phosphatase and tensin homologue (*PTEN*) (Cowden syndrome) (12, 13). Over 90% of women with EC present with postmenopausal bleeding (PMB), defined as bleeding occurring at least a year after cessation of menstruation due to menopause (14). Only 5%–10% of women with PMB, however, will have EC, but the risk increases with age and in the presence of other risk factors (15). Premenopausal and perimenopausal women may present with irregular or heavy menstrual bleeding (14). Abnormal vaginal discharge, hematuria, pelvic pain, or pain during sexual intercourse are other important but less common symptoms (16).

Treatment for EC is primarily surgical with hysterectomy and bilateral salpingo-oophorectomy as standard of care worldwide (14, 15). Women with high-risk disease are offered adjuvant radiotherapy and/or chemotherapy to reduce the risk of recurrence (17, 18). Women with advanced (stages III and IV) or metastatic EC have a poor prognosis (<20% 5-year survival) and are at a higher risk of relapse compared to those diagnosed early (>90% 5-year survival) (14, 15). There are limited evidence-based treatment options available for women diagnosed at a late stage; thus, it is crucial that women are diagnosed early when treatment is able to effect cure. Early detection will also allow for radical treatments to be minimized and enable conservative management options to be offered to women of child bearing age and those with morbid obesity in whom surgery is potentially hazardous (15).

## The Diagnosis of Endometrial Cancer

The diagnostic strategy for suspected EC has not evolved in several decades; yet, it is far from perfect. In the United Kingdom, the National Institute for Health and Care Excellence (NICE) recommends that women aged 55 and over with PMB be referred to the rapid access gynecology clinic to be seen within 2 weeks (14, 19, 20). Such a strategy could miss cases of EC, so NICE also recommends consideration of a 2-week wait referral for those aged under 55 with PMB, as well as direct access ultrasound for a select group of women aged 55 and over with unexplained vaginal discharge or frank hematuria (19, 20). Transvaginal ultrasonography (TVS) is the imaging modality of choice for the initial evaluation of suspected EC (5). Measurement of endometrial thickness (ET) using TVS is non-invasive, precise and sensitive, and is particularly useful when the endometrium is homogenous (21). Its diagnostic utility for EC is, however, limited by a low specificity, as a thickened endometrium may be caused by other pathologies including endometrial polyps, intracavitary fibroids and artefacts such as blood clots, and is seen in approximately 50% of women undergoing TVS for suspected EC (21–23). Women with a thickened endometrium therefore require further invasive tests in order to establish a diagnosis. Endometrial sampling has good accuracy for EC detection and is the gold standard for the diagnostic evaluation of women with suspected EC (5). It may, however, miss focal pathologies including EC, especially when performed blindly, as less than 50% of the endometrium is usually sampled (24). In addition, the procedure can be painful, especially in nulliparous women, and the risk of failure is high (25). Hysteroscopy with targeted biopsy is indicated following failed endometrial sampling or in cases of an irregular endometrium in the presence of risk factors, but is invasive and often plagued by operative challenges (15). In the outpatient setting, pain, cervical stenosis and sub-optimal visualization of the uterine cavity are the most frequent reasons for abandonment of procedure (14). Rarely, life threatening complications such as uterine perforation and cervical laceration occur (26).

While invasive testing is necessary for a tissue diagnosis, most women with PMB have a benign explanation for their bleeding. Currently, thousands of women with PMB undergo hysteroscopy and/or endometrial biopsy, invasive tests that are unpleasant, sometimes technically challenging, and painful or extremely painful for 30%–40% of women (15, 26, 27). Restricting invasive testing to women with sinister underlying pathology would save many thousands of women per year in every developed country in the world from invasive tests they do not need. A novel EC detection tool that could triage women for invasive testing or quick reassurance would transform clinical pathways for EC.

The ideal detection tool is simple, non-invasive, accurate and cost effective. It should be able to identify women with EC at the earliest possible stages while re-assuring the large majority who do not have EC (28). “What simple, non-invasive, painless, cost-effective, and convenient tests can be used to detect cancer early?” ranked first in the top ten research priorities for early cancer detection by the James Lind Alliance partnership representing patients, carers, and clinician groups (29). A similar study exploring unmet research needs in EC, found “which women with abnormal bleeding require urgent specialist referral and which can be safely reassured” to be second most important priority (30). Based on these gap analyses, studies exploring EC detection using non-invasive samples such as urine are urgently needed (28).

## Biomarker Discovery, Validation, and Clinical Utility

A biomarker is defined by the National Cancer Institute as a “biological substance in body fluids or tissues that is indicative of a normal or abnormal process or of a condition or disease” (31). Biomarkers can be proteins and peptides (e.g., an enzyme or receptor), nucleic acids (e.g., DNA, microRNA), antibodies or metabolites. Biomarkers can have single or multiple components, for example, individual proteins (e.g., CA-125) or genomic, proteomic or metabolomic signatures (32).

Multiple approaches have been employed in the search for cancer diagnostic biomarkers. A classic approach is to select potential markers based on tumor biology. More recently, however, with the advent of new technologies including next-generation sequencing and mass spectrometry (MS), an objective and pragmatic approach to biomarker identification using biofluids has come to the fore (32). Potential diagnostic biomarkers must overcome several hurdles before they can be used in the clinical setting: discovery, validation, and verification (32, 33). Importantly, the performance of any novel test needs to be evaluated in terms of its analytical performance, clinical validity and clinical utility (**Table 1**) (34). Analytical performance refers to the accuracy with which a particular characteristic of interest can be identified by a given laboratory test. An ideal biomarker assay should not only be accurate but also reproducible within and between laboratories. The accuracy with which a test identifies a patient’s clinical status such as the presence of EC (clinical validity) and the risks and benefits resulting from the test use (clinical utility) are other test properties that must be considered. Clinical validity is described in terms of sensitivity, specificity, positive predictive value (PPV) and negative predictive value (NPV) (**Table 1**) (34, 35). Safety, acceptability, fit in the diagnostic pathway, and cost effectiveness inform clinical utility and must be taken into consideration when evaluating a novel diagnostic test prior to translation into routine clinical settings (**Table 1**) (35, 36).

**Table 1:** Characteristics of the optimal EC detection tool.

Considerations	Definition	Formula	Implications in EC	Ideal EC diagnostic test criteria
**Sensitivity**	Probability that a person with a disease will test positive	TP TP + FN	A low sensitivity would mean a large proportion of women with EC will be falsely re-assured leading to delayed presentation (the false negatives who will later present at an advanced stage) and poor survival.	Maximal sensitivity (100%)
**Specificity**	Probability that a person without a disease will test negative	TN TN + FP	A low specificity would mean a large proportion of women without EC will undergo further unnecessary & invasive tests/treatments. The worried well population also increases.	Maximal specificity (100%)
**Positive predictive value** **(PPV)**	Probability that a positive test will correctly identify those with the disease	TP TP + FP	A low PPV has implications for women with a positive test, a large proportion of whom will undergo further unnecessary diagnostic tests or even treatments that are not indicated.	Maximal PPV (100%)
**Negative predictive value** **(NPV)**	Probability that a negative test will correctly identify those without the disease	TN TN + FN	A low NPV has implications for women with a negative test, a large proportion of whom will be falsely reassured.	Maximal NPV (100%)
**Clinical Utility**	Risks and benefits resulting from test use.	Rates of acceptability, complications and side effects	A highly invasive test is less acceptable to patients and may lead to complications.	Safe, minimally invasive, sensitive and specific, acceptable, minimal side effects
**Cost Effectiveness**	Direct monetary costs and indirect costs associated with the disease, tests and a misdiagnosis of the disease	Cost Effectiveness Ratio CER: Cost of intervention Effect of intervention	An expensive test is unlikely to be affordable by patients or health service providers including the NHS	Cheap/cost effective

TP, True Positives; TN, True Negatives; FP, False Positives; FN, False Negatives.

## Urinary Biomarkers for EC Detection

Urine is the prototype non-invasive sample and is a useful biological fluid for biomarker discovery due to its accessibility and potential for repeated samples and unlimited volumes. Its collection is cheap and is usually without side effects or complications (37). Thus, it fits the description of an ideal biomarker source for EC detection (**Table 1**). A wide variety of substances with potential to serve as EC biomarkers can be found in urine and include endogenous metabolites, genetic products such as tumor DNA, peptides/proteins, malignant cells and secreted organelles such as extracellular vesicles (38–40). The exploration of each of these targets for biomarker identification requires the use of specialized techniques based on platforms such as cytology/single cell technology, spectroscopy, genomics, transcriptomics, proteomics, and metabolomics (33, 41, 42).

Identification of urinary biomarkers for endometrial cancer detection relies on the excretion of systemic biomarkers by the kidneys or urinary contamination with biomarkers shed from the uterus (28, 43). Systemic proteins and metabolites excreted in urine originate from several organs including the uterus and find their way into the proximal tubules by escaping total reclamation by the renal filtration barrier (44). Proteins and peptides excreted in urine are less complex and more stable compared to plasma proteins, thus conferring an advantage for biomarker discovery (37).

The anatomical continuity between the upper and lower genital tracts provides an opportunity for non-invasive sampling of uterine derived proteins and malignant cells (45). Previous studies have shown that endometrial tumor debris passes through the cervix and into the vagina, from where it can be collected with soft brushes or tampons (46–48). The proximity of the urethra to the vagina also allows for the potential contamination of self-collected urine by naturally shed tumor material. While renally excreted biomarkers may be difficult to measure due to the low abundance of tumor-derived molecules in the circulation, especially in early stage EC, for uterine shed biomarkers, it is the consistency and reliability with which these contaminate urinary samples that limits their clinical utility, especially in asymptomatic women.

**Figure 1:**
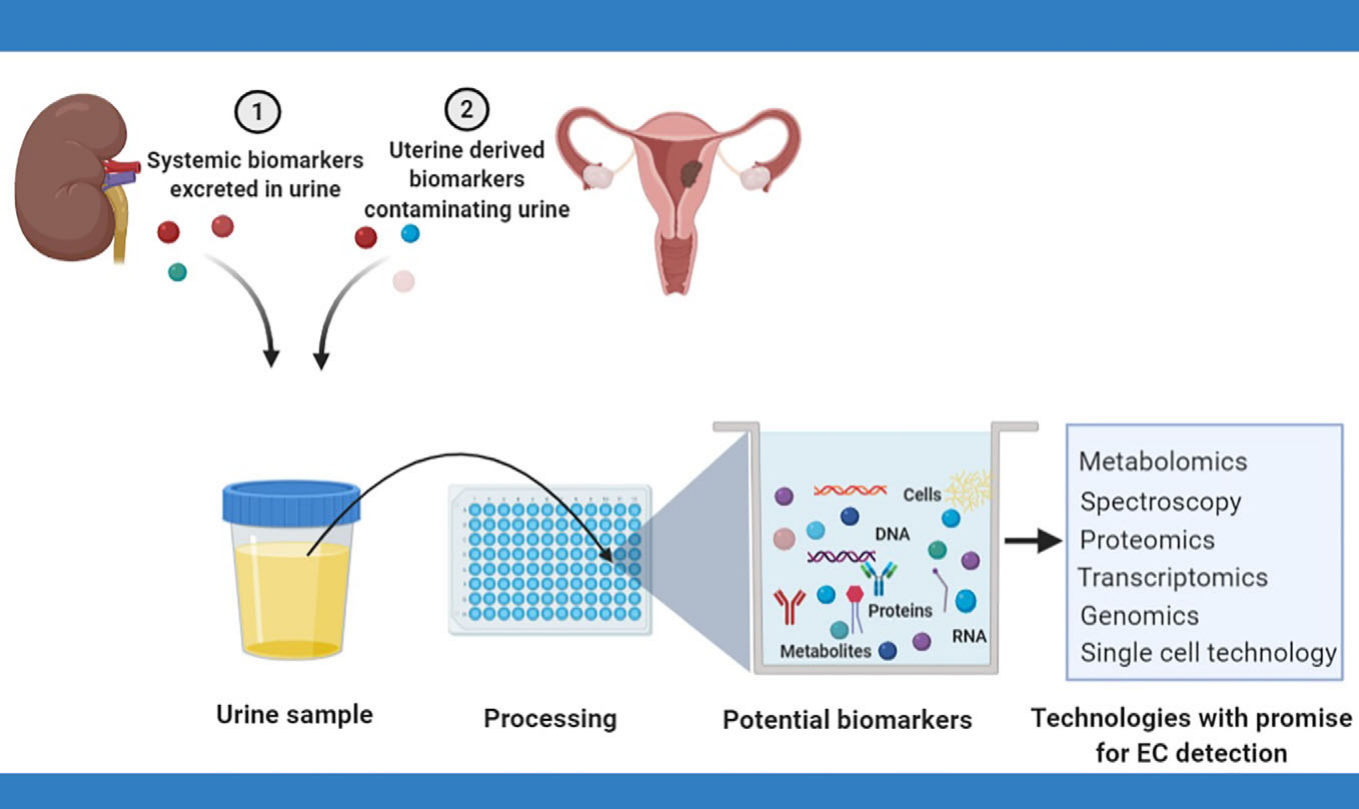
Urinary biomarkers for endometrial cancer detection rely on the renal excretion of systemic biomarkers or the contamination of urinary flow by naturally shed uterine biomarkers. Several techniques have potential for EC biomarker discovery and include cytology, spectroscopy, metabolomics, transcriptomics, and proteomics.

Techniques for cancer diagnosis based on urine analysis have evolved over time from the microscopic assessment of urinary sediments to the comprehensive examination of urinary analytes, made possible by recent advances in high-throughput technologies (42). Endometrial cancer cells may be identified in urine, especially in women with bleeding symptoms, by the microscopic assessment of urine (cytology) (39) or by the use of single cell technology (49). Urinary cell-free tumor DNA, on the other hand, may be renally excreted or may result from the breakdown of malignant cells contaminating urinary flow. Tumor DNA characterization, including an assessment of DNA concentrations, the presence of mutations and methylation status in urine has great potential to yield relevant biomarkers and needs further exploration. Urinary proteins and micro-RNA closely mirror the dynamic state of cells and are viable sources of EC biomarkers (28). Metabolites, on the other hand, are the most proximal of the omics markers and best reflect a cell systems physiological phenotype (42). **Figure 1** summarizes the various sources of urinary biomarkers for EC detection and the technologies with promise for EC urinary biomarker research.

### Endogenous Urinary Metabolites

Metobolomics has been employed to analyze urine for EC biomarkers (see **Table 2**). This is because it systematically identifies and quantifies metabolic products from cells, tissues or biofluids (65). By enabling the analysis of the downstream products of genomic, transcriptomic and proteomic processes, metabolomics closely mirrors a systems phenotype and effectively summarizes the effects of other “omics” technologies (42). Metabolic profiling can either be targeted or untargeted. While targeted approaches deal with the measurement of a pre-defined select group of metabolites, untargeted approaches aim to comprehensively analyze all measurable products in a given sample with no prior assumptions (42, 66, 67). The hypothesis driven nature of targeted studies lends to a high level of precision and accuracy, in contrast to untargeted approaches which are prone to false positives (42). Targeted approaches are thus often employed to validate findings obtained from untargeted studies. Two platforms are commonly used in metabolomic biomarker research: MS and nuclear magnetic resonance (NMR) spectroscopy (33, 68).

**Table 2:** Study characteristics and diagnostic accuracies of potential urinary biomarkers for EC detection.

Study Title	Type of marker	Marker(s)	Test platform	Study design	Urine collection	Country
**Shao et al. (** 50 **)**	Metabolites	Porphobilinogen & acetylcysteine were downregulated while N-acetylserine, Urocanic acid & Isobutyrylglycine were upregulated. Diagnostic model: 82.29% accuracy	Metabolomics: Ultra-performance liquid chromatography (LC) quadrupole time of flight mass spectrometry (MS).	Case control design: 25 EC cases, 25 healthy controls and 10 EH cases.	Morning urine collected a day before surgery for cases. Similar sample from healthy controls	China
**Zhao et al. (** 51 **)**	Metabolites	4-hydroxyestradiol was upregulated in EC while 2-methoxyestrone and 2-methoxyestradiol were downregulated	Metabolomics: Liquid chromatography-mass spectrometry with hollow fiber liquid-phase microextraction.	Case control design: 23 pre-operative post-menopausal women with EC (cases) and 23 post-menopausal healthy controls.	24-h urine samples collected in 1-L bottle containing 1g of ascorbic acid.	China
**Bufa et al. (** 52 **)**	Metabolites	Several steroid metabolites including androsterone, etiocholanolone, 11beta-hydroxy-androsterone were downregulated in EC versus controls	Quantitative real time polymerase chain reaction (PCR)	Case control study: 12 EC cases and 10 age-matched controls.	24-h urine samples	Hungary
**Aitokallio-Tallberg et al. (** 53 **)**	Metabolites	6-keto-prostgalndin F1a: No difference found between EC cases and controls	Radioimmunoassay and High performance liquid chromatography	Case control design: 12 EC cases, other cancers, 12 control women.	Pre-operative spontaneous void urine samples	Finland
**Mu et al. (** 38 **)**	Proteins	Zinc alpha-2 glycoprotein, alpha1-acid glycoprotein and CD59 had altered levels in EC cases versus controls.51- kDa of nebulin was down regulated in EC.	Proteomics: Two-dimensional gel electrophoresis and o-glycan binding lectin & LC-MS/MS	Case control design: 7 cases with newly diagnosed EC stages 1B and IIA/B. 11 age-matched healthy controls.	Morning void.	Malaysia
**Mu et al. (** 54 **)**	Peptides	Glycopeptides with mass/charge ratio of 1449 could differentiate EC from ovarian and cervical cancers	Proteomics: Surface enhanced laser desorption/ionization-time-of-flight (SELDI-TOF)	Case control design: 4 EC, 4 ovarian and 4 cervical cancer cases.4 healthy volunteers as controls.	50-ml morning midstream urine	Malaysia
**Bostanci et al. (** 55 **)**	Proteins	Neopterin was upregulated in EC cases compared to controls	High performance Liquid chromatography-	Case control: 41 EC cases and 41 healthy controls	Not specified	Turkey
**Bazzett et al. (** 56 **)**	Proteins	Matrix metalloproteinases (MMP): No association between EC and urinary MMP	Gel Electrophoresis, western blot with anti-MMP antibodies	Case control design: 31 EC cases, 19 controls. Also had 29 ovarian, 31 cervical and 5 vulvar CA cases.	Clean catch void. Samples that tested positive for blood were excluded.	United States
**Mattila et al. (** 57 **)**	Proteins	Epidermal growth factor (EGF): Immunoreactive EGF was upregulated in urine of EC patients.	Radioimmunoassay and gel exclusion chromatography	Case control: EC cases, other cancers and age and sex-matched controls.	Spot urine, otherwise non-specified.	Finland
**Stockley et al. (** 58 **)**	Proteins	Urine sediment MCM5 discriminated EC from benign disease with AUC of 0.83. At 12pg/mL, sensitivity was 87.8% and specificity 75.9%	Enzyme Linked Immunosorbent Assay (ELISA)	Case control design: 41 EC, 58 benign gynecological controls, 26 ovarian cancer.	Full void urine	United Kingdom
**Zavesky et al. (** 40 **)**	Cell free micro-RNA	miR106b was down regulated in EC cases compared to controls.	Quantitative real time polymerase chain reaction (PCR)	Case control study: 10 EC cases, other cancers, healthy controls	Second morning void	Czech republic
**Zavesky et al. (** 40 **)**	Exosome RNA	No significant de-regulation in micro-RNA was found	Urine exosome isolation kit, PCR	Case control study: 10 EC cases, other cancers, healthy controls	Second morning void	Czech republic
**Ruskin et al. (** 59 **)**	Exosome RNA	Ten micro-RNA (has-miR-155-5p, has-miR-425-5p, has-miR-23a-3p, has-miR-21-5p, has-miR-200c-3p, has-miR-124-3p, 100-5p,26a-5p, has-miR-99a-5p has-miR-,181a-5p) had at least 30 fold higher expression in EC and two had at least two fold reduced expression.	Urine exosome isolation and purification kit, real time PCR	Case control study: 12 EC cases and 5 controls.	Not specified	United States
**Srivastava et al. (** 60 **)**	Exosome RNA	has-miR-200c-3p was differentially expressed between EC cases and controls.	Urine Exosome isolation, microRNA PCR array	Case control study: 22 EC cases and 5 symptomatic controls	30–50mils urine collected under sterile conditions in operating suite prior to surgery	United States
**Kinugasa et al. (** 61 **)**	Hormone	B-core fragment of human chorionic gonadotropin: Elevated levels found in 37.8% (14 of 37 EC cases). Levels still low for EC detection	Enzyme immunoassay, gel chromatography	Case control: 37 EC cases, other cancers	Not-specified.	Japan
**Wang et al. (** 39 **)**	Cytology	Atypical squamous cells in the urine in a symptomatic patient (hematuria)	Urine cytology	Case report	Not specified	United States
**Kanno et al. (** 62 **)**	Cytology	Positive urine cytology in 76 year old with EC presenting with gross hematuria	Urine cytology	Case Report	Gross hematuria	Japan
**Khan et al. (** 63 **)**	Cytology	Positive urine cytology in a 66 year old with microscopic hematuria	Urine cytology	Case report	Urine sample with microscopic hematuria	United Kingdom
**Paraskevaidi. (** 64 **)**	Spectroscopy	Biomarker algorithm with 95% sensitivity and 100% specificity	Total reflection Fourier-transform infrared (ATR-FTIR) spectroscopy	Case control study: 10 EC, 10 Ovarian and 10 healthy controls.	Pre-operative catheter urine specimens obtained after at least 6 h of fasting	United Kingdom

Multiple studies have sought to identify possible urinary metabolites with potential for EC detection (50, 51, 53). Amino acid, lipid and hormonal metabolites have all been suggested to have potential as EC diagnostic biomarkers. Shao and colleagues, using ultra-performance liquid chromatography quadrupole time-of-flight MS (UPLC-Q-TOF/MS) on urinary specimens from 25 EC cases and 25 controls identified a set of five metabolites as possible biomarkers for EC detection: porphobilinogen, acetylcysteine, N-acetyserine, urocanic acid, and isobutyrylglycine (50). Of these five, porphobilinogen and acetylcysteine were downregulated in EC while N-acetyserine, urocanic acid and isobutyrylglycine were upregulated (50). A predictive model based on these five biomarker candidates using the partial least squares-discriminant analysis was able to distinguish EC from endometrial hyperplasia (EH) (n = 10) and healthy controls. While porphobilinogen and acetylcysteine discriminated between EC and the merged group of EH and healthy controls, there was no significant difference between EH and healthy controls (50). None of these biomarker candidates have been independently validated and further studies are needed to elucidate their role in EC tumorigenesis. Some urinary metabolites have also been reported as being able to discriminate between EC (n = 40) and benign ovarian tumors (n = 62). These include 3-dehydroquinic acid, 3-indolelactic acid, S-reticuline, selenocystathionine, 1-(1Z-hexadecenyl)-sn-glycero-3-3-phosphate, N-acetylneuraminic acid, 3-sialyl-N-acetyllactosamine and 3-sialylactose (42).

Zhao and colleagues investigated endogenous estrogen metabolites as biomarker candidates for endometrial cancer diagnosis using urine samples from 23 EC cases and 23 post-menopausal healthy controls (51). While 4-hydroxyestradiol (4-OHE2) was up-regulated in EC, 2-methoxyestrone (2-MeOE1) and 2-methoxyestradiol (2-MeOE2) were down-regulated. Twenty-four-hour urinary 17β-estradiol (E2) was also found to be elevated in EC cases (51). E2 is linked to endometrial carcinogenesis through the activation of P13K/AKT and MAPK signaling pathways. 4-OHE2, on the other hand, has been linked with EC tumorigenesis through the upregulation of CYP1B1 (51). 2-MEOE1, an estrone analogue of 2-MeOE2, exhibits anti-proliferative and pro-apoptotic properties, in keeping with the finding of its down-regulation in EC (51, 69). While hormonal imbalance from adipose derived unopposed estrogen is the most established biological pathway implicated in obesity driven endometrial carcinogenesis, the finding of endogenous estrogen metabolites in urine is not necessarily diagnostic of EC (70). They do, however, provide unique insights in endometrial cancer urinary biomarker discovery and may, in combination with other biomarker candidates, be used to improve the accuracy of an EC urinary biomarker panel. Other approaches that have been tried include the use of attenuated total reflection Fourier-transform infrared (ATR-FTIR) spectroscopy by Paraskevaidi and colleagues, who analyzed urinary specimens form 10 EC cases, 10 ovarian cases and 10 healthy controls (64). They were able to develop a biomarker algorithm with 95% sensitivity and 100% specificity for EC detection that is yet to be externally validated (64). Several other studies (52, 53, 61) have explored metabolites for EC detection in urine, however, none have yet been translated into routine clinical use.

A number of issues need to be addressed when developing robust urine-based metabolite biomarker discovery protocols (**Table 3**). First, is the variability of exogenous sources of urinary metabolites. While the endogenous metabolic process is prone to individual biological variations, there is greater variability in the metabolic products resulting from exogenous substances such as water, drugs and food and this can significantly impact on study findings (**Table 1**). As such, it is important to identify these potential confounding variables and control for them (**Table 3**). The collection of urine after an over-night fast, for instance, can control for diet and is encouraged (67). Seasonal variations in dietary and other lifestyle factors such as levels of physical activity can be minimized by ensuring that urine samples are collected at a specific time of the year and not all year round (42). Strategies often used to control for drug effects include sample collection before any medications are used on the day, asking study participants to temporarily withhold use of medications where feasible and excluding specific drug metabolites during analysis (42). Levels of several urinary metabolites also exhibit a circadian rhythm (76). As such, standard operating procedures should be applied, especially with regards to time of sample collection (28). With obesity as the strongest risk factor for EC (70), urinary metabolic markers of adiposity are likely to systematically differ between EC cases and controls and should be controlled for (74). Such markers may be used in combination with other biomarker candidates to improve their diagnostic accuracy and address the issues shown in **Table 1** with respect to false positive results.

**Table 3:** Important considerations in the design of EC urinary biomarker studies.

Considerations	Role in EC pathogenesis and risk	Effect on urine biomarker research	Control strategy
**Age**	EC is a disease of the elderly, 2/3^rd^ of all cases are diagnosed between ages 50 and 74 (14)	Age related changes in urinary protein excretion. Several metabolites are linked to the ageing process (71)	Age group eligibility criteria Balance in age distribution between cases and controls Co-variant analysis (42)
**Diet**	Evidence linking diet and brewed drinks including isoflavone (soy), coffee, and tea to EC risk (10). Possibility of differential use between cases and controls.	Exogenous source of metabolites, prone to individual variability, can confound biomarker findings (42).	Urine collection after an overnight fast Co-variant analysis (42)
**Medications**	Use of medications linked to conditions that can increase EC risk such as hypertension may systematically differ between EC cases and controls (14, 72),	Linked to urinary protein and metabolic profile. Anti-hypertensive can influence urinary proteome (73), thus confounding biomarker findings, individual variability in their use (42).	Urine collection before specific drug intake on the day if feasible, Asking participants to withhold drug use temporarily if feasible Exclusion of drug related metabolites during analysis Co-variant analysis
**Physical activity level/BMI**	High levels of physical activity reduces EC risk (10). High BMI increases risk of EC (70). Possibility of differential BMI between EC cases and controls	Urinary metabolic markers of adiposity likely to differ between cases and controls (74). Physical activity impacts on urinary protein levels.	Co-variant analysis (42). Exclusion of urinary markers of adiposity Balance in median BMI between study arms
**Menopausal status**	EC is mostly a post-menopausal disease (28)	Hormone altering conditions like menopause may influence urine metabolic profiles (42)	Exclusion of pre-menopausal women Balance in proportion of pre/postmenopausal women between cases and controls. Co-variant analysis (28)
**Smoking**	Smoking reduces EC risk (10). Possibility of differential use between cases and controls.	Urinary nicotine metabolites (75) may differ between EC cases and controls	Co-variant analysis (42) Balance in proportion of smokers in study arms Exclusion of nicotine metabolites.
**Geographical location**	EC is more common in Western countries compared to developing countries. Important in cross-national studies	Geographical variation in lifestyle factors that can influence urinary metabolic and urinary profiles (42).	Urine collection from participants from a homogenous region. Co-variant analysis (42)
**Seasonal effects**	Not applicable	Evidence of seasonal effects of diet, lifestyle and exercise patterns on metabolic urinary profiles (42).	Urine collection at a specific time of the year and not all year round (42). Co-variant analysis

### Urinary Proteins and Peptides

Protein and peptide profiling are important tools for biomarker discovery and have been explored in various studies seeking to identify urinary biomarkers for EC detection. Proteomics systematically characterizes all of the proteins within a cell and in combination with computational analyses and machine learning techniques is able to identify biomarkers that differ between cohorts (28). Using 2-dimensionalgel electrophoresis (2-DE) and O-Glycan binding lectin analysis by LC-MS/MS, Mu and colleagues demonstrated altered levels of Zinc alpha-2 glycoprotein (ZAG), alpha-1 acid glycoprotein (AAG) and CD59 in the urine specimens of EC cases compared to healthy controls. Nebulin, on the other hand, was found to be downregulated (38). These biomarkers, though promising, are limited by their lack of specificity for EC and are yet to be validated. Importantly, 2-DE is a time consuming technique with limited sample throughput and narrow depth of proteome analysis. Bostanci and colleagues used high performance liquid chromatography to compare urine samples from 41 EC cases and 41 healthy controls and found neopterin to be differentially expressed (55). Neopterin, a marker of cellular immune activation, was also able to differentiate early from late stage disease, suggesting a potential prognostic use especially if it is found to be a specific marker of EC and not an inflammatory response marker. The positive predictive value of neopterin is poor, however, as elevated levels may also be found in autoimmune conditions, viral and bacterial infections and other malignant states (55). In the study by Stockley and colleagues, urine sediment MCM5 discriminated EC cases (n = 41) from benign gynecological disease (n = 58) with an AUC of 0.83. At a 12pg/ml cut-off value, a sensitivity of 87.8% was reported at 75.9% specificity for EC exclusion (58). The finding of MCM5, a marker of cellular proliferation, in the urine sediments of EC cases, is most likely reflective of the contamination of urine by uterine shed cellular components/proteins, rather than the renal excretion of systemic proteins. However, this finding is yet to be externally validated and further studies are needed. The upregulation of MCM5 has been reported in several other cancers, including bladder and cervical cancers, thus limiting its utility as a specific marker for EC detection.

A number of proteins have been suggested as potential biomarkers for EC detection based on proteomic analysis of blood (28). Although none have been translated into routine clinical practice due mainly to their sub-optimal accuracy and lack of robust validation, the results are encouraging, especially with the development of multi-marker panels and integration of clinical, genomic, transcriptomic and metabolomic data. There is, however, insufficient evidence to suggest that the urinary equivalents of the blood-based EC biomarker candidates are as promising. As an example, serum epididymis protein 4 (HE4), a useful biomarker in the management of ovarian cancer has been reported as a potential biomarker for EC detection (77–79). While multiple studies suggest a potential utility of urine HE4 in discriminating ovarian cancer cases from the general population, there is a lack of evidence to suggest a similar utility for EC detection (37). Similarly, Matrix Metalloproteinases (MMP) have been reported as blood based EC biomarker candidates (28). However, Bazzett and colleagues, using gel electrophoresis on urine samples from 31 endometrial cancer cases and 19 controls found insufficient evidence to recommend urinary MMP for EC detection (56). Mattilla and colleagues, on the other hand, when investigating urinary epidermal growth factor concentrations in various human malignancies found an up-regulation of immunoreactive EGF in the urine samples of EC cases (57). EGFR is a receptor tyrosine kinase that is over expressed in several human malignancies including EC where a 46%–67% expression rate has been reported. Further studies are needed to validate its potential utility for EC detection.

Urinary glycosylated peptides have also been suggested as potential EC biomarkers. Using SELDI-TOF in an N-glycopeptide profiling of urine samples from patients with EC, ovarian and cervical cancers, Mu et al. demonstrated that urinary glycopeptide with mass/charge ratio 1449 was able to differentiate EC from ovarian and cervical cancers (54). This was however based on a small number of subjects and it was unclear whether the study was sufficiently powered for biomarker identification. Certainly, SELDI-TOF offers little in terms of biomarker characterization compared to more recent technologies.

The most important consideration in EC urinary protein biomarker research is the wide variability in urinary protein concentrations due to factors such as age, genetics, physical activity status, drugs and diet among others (**Table 3**) (37, 73). Hypertension, for instance, is a risk factor for EC and the prevalence of hypertension, and by extension, the use of anti-hypertensives, is likely to systematically differ between cohorts of women with and without EC (44). Strategies to deal with such confounding variables are summarized in **Table 3**.

### Urinary Cell-Free and Extracellular Vesicle microRNAs

MicroRNAs are small noncoding RNAs with post-translational regulatory functions in a wide variety of cellular processes including tumorigenesis (80, 81). They have emerged as viable biomarker candidates for cancer diagnosis and have been explored in various studies using tissue specimens and cell lines. In recent times, research focus has shifted to identifying microRNAs in minimally invasive samples such as plasma/serum (82). Available evidence suggests that cell free micro-RNAs may be detected in a wide variety of non-invasive bodily fluids including urine (40). The diagnostic potential of urinary cell free microRNAs has been explored, particularly in upper urinary tract and bladder urothelial cancers. Zavesky and colleagues, using quantitative real-time PCR, investigated the expression of candidate cell-free urinary microRNAs in endometrial and ovarian cancer patients and reported miR106b to be downregulated in EC cases compared to controls (40).

Urinary extracellular vesicles have also been explored as possible sources of microRNA for EC diagnosis. Using exosome RNA, Zavesky and colleagues found no significant de-regulations in microRNA expressions in EC versus controls (40). Ruskin et al., on the other hand, isolated urinary exosomes from 12 EC cases and 5 controls and reported 10 microRNAs to have at least 30 fold higher expression in EC while two microRNAs had at least a two-fold reduced expression (59). While further studies are very much needed to validate these biomarker candidates, the results offer cautious optimism as three of the overexpressed microRNAs had *PTEN*, Notch, Vascular Endothelial Growth Factor (VEGF), Protein Kinase B (AKT), and Programmed Cell Death 4 (PDCD4) as molecular targets. *PTEN*, a tumor suppressor gene that negatively regulates PI3-AKT signaling pathway has been implicated in endometrial carcinogenesis (83), as has Notch, a regulator of cellular proliferation, differentiation and apoptosis (84). Angiogenesis has been shown to play an important role in the growth of EC (85) while PDCD4 expression has been linked to tumor grade in endometrioid EC (86). Srivastava and colleagues studied 81 microRNAs from urine derived exosomes from patients with and without EC, 57 of which were amplified in qPCR and reported has-miR-200c-3p to be differentially expressed (60). miR-200c is a tumor suppressor microRNA that prevents the epithelial to mesenchymal transition of cancer cells and has been found to be dysregulated in many cancers (87). Its utility for EC detection is thus limited by its low specificity. While microRNA profiling of endometrial cancer is yet to be fully characterized, there is evidence in support of the differential expression of miR-200 family of microRNAs in endometrial cancer tissues in comparison to normal endometrium (60). Urinary miR-200c3p is thus a promising non-invasive EC biomarker but needs validation (as part of a microRNA panel) prior to translation into routine clinical practice. Other microRNA candidates and the estimates of their differential expression are summarized in **Table 2**.

### Tumor Cell Identification

Identification of malignant cells in urine is a potential EC diagnostic strategy that needs exploration, especially in symptomatic women. Bladder cancer can be detected by microscopic evaluation of exfoliated urothelial tumor cells (88, 89). Wang and colleagues report on the role of urine cytology in detecting EC following the presentation of an 82 year old woman with hematuria (39). The possibility of finding endometrial malignant cells in urine is dependent on its contamination by EC cells shed during postmenopausal bleeding since the local infiltration of the bladder by metastatic disease is a late and rare event in EC (63). A papillary structuring of malignant clusters of cells in urine has been reported in both transitional cell carcinoma of the bladder and serous endometrial cancer (63). Positive urine cytology in women with hematuria, in the absence of a demonstrable urinary tract malignancy, should therefore raise the suspicion of a possible uterine cancer. Intermittent tumor shedding may not lend itself to reliable biomarker detection, particularly in women without bleeding symptoms; however, very little research has been done (28). In the UK, the NICE recommends a direct access ultrasound scan to assess for EC in women aged 55 and over who present with visible hematuria with either low hemoglobin levels, thrombocytosis or high blood glucose levels (20). Urine cytology may offer further evidence for a likely endometrial malignancy, especially if it is shown to out-perform TVS in terms of accuracy. Cytology is, however, limited by its dependency on the experience of the cytologist which may vary widely. Platforms such as single cell technology have shown great promise in identifying circulating tumor cells in bodily fluids but are limited by costs and experimental time (49). Flow cytometry is another technology that has been explored in identifying cancer cells in urine (90). Further studies are needed to assess the accuracy of tumor cell identification in the urine of symptomatic women with EC, especially those who present with hematuria (28).

## Conclusion

A urine-based biomarker is ideal for EC detection and relies on the renal excretion of systemic biomarkers or urinary contamination with biomarkers shed from the uterus. Urinary metabolites, proteins and micro-RNA have all been reported as possible EC biomarker candidates. Currently, there is insufficient evidence to support their use due to lack of robust validation. Most of the studies exploring urine for EC diagnosis have been small pilot studies and larger studies are needed. While systemic biomarkers excreted in urine are somewhat limited by the low abundance of cancer related signals in the circulation, especially in early stage EC, contaminating biomarkers in the urine from the uterus may be unreliably shed, especially in asymptomatic women. Molecular analysis of urinary specimens from symptomatic women with EC has great potential to yield clinically relevant non-invasive biomarkers for EC detection. Spectroscopy and tumor cell identification by cytology or single cell technology are other diagnostic strategies with promise that need further exploration, especially in symptomatic women. The use of artificial intelligence to combine “signals” from several modalities or those found using a specific technique will benefit the discovery and validation process. Several factors including age, diet, level of physical activity, medication use, menopausal status, and seasonal effects must be taken into consideration when designing studies for urinary EC biomarker discovery.

## Author Contributions

Conceptualization: KN. Writing—original draft preparation: KN. Writing—review and editing: KN, DC, ERJ, HO’F, CEB, EJC, and ADW Visualization: KN, DC, ADW, and EJC. Supervision: DC, ADW, and EJC. Funding acquisition: ADW and EJC. All authors contributed to the article and approved the submitted version.

## Funding

KN is supported by a Cancer Research UK Manchester Cancer Research Centre Clinical Research Fellowship (C147/A25254) and the Wellcome Trust Manchester Translational Informatics Training Scheme. HO’F is supported by the National Institute of Health Research Doctoral Research Fellowship (DRF-2018-11-ST2-054). ERJ is supported by a grant from the JP Moulton Charitable Foundation. EJC and ADW are supported by NIHR Manchester Biomedical Research Centre (IS-BRC-1215-20007). The Medical Research Council (MR/M008959/1) and CRUK Manchester Major Centre award (C147/A25254) supports work in Whetton’s Lab. CEB is funded on a Manchester University NHS Foundation Trust Clinical Research Fellowship.

## Conflict of Interest

The authors declare that the research was conducted in the absence of any commercial or financial relationships that could be construed as a potential conflict of interest.
